# Trajectories of Insomnia Symptoms Among Aging Employees and Their Associations With Memory, Learning Ability, and Concentration After Retirement - A Prospective Cohort Study (2000–2017)

**DOI:** 10.1177/08982643221078740

**Published:** 2022-04-28

**Authors:** Antti Etholén, Olli Pietiläinen, Anne Kouvonen, Mirja Hänninen, Ossi Rahkonen, Tea Lallukka

**Affiliations:** 1 Department of Public Health, 176449 University of Helsinki, Helsinki, Finland; 2 Faculty of Social Sciences, 176449 University of Helsinki, Helsinki, Finland; 3 Centre for Public Health, Queen’s University Belfast, Belfast, UK

**Keywords:** insomnia symptoms, cognitive function, retirement

## Abstract

**Objectives:**

We applied a person-oriented approach and used latent class linear mixed models to identify sleep trajectories that explain memory, concentration, and learning ability problems after retirement.

**Methods:**

Data consist of prospective surveys from four phases of the Helsinki Health Study between 2000–2017 (n = 3748, aged 55–77 years, 80% women). Multinomial regression was used to examine the associations between sleep trajectories and cognitive function, adjusting for sociodemographic, health-related behavior, and health factor covariates.

**Results:**

Among statutory retirees, three latent group trajectories of insomnia-related symptoms were identified: stable low, decreasing, and increasing. Among those who had retired for disability reasons, we identified one additional latent group trajectory: stable high. Insomnia symptoms were associated with worse cognitive function.

**Discussion:**

Early detection of insomnia symptoms would be a potential intervention point to improve both sleep quality and prevent cognitive decline in later life. However, intervention studies are needed.

## Introduction

Insomnia symptoms are commonly experienced at all ages but especially among older adults (Ohayon et al., 2004). With aging, cognitive function typically weakens and slows down (Roberts & Allen, 2016). Both cognitive decline and insomnia are associated with poorer quality of life (Christiansen et al., 2019; Stites et al., 2018; Kyle et al., 2010). Sleep efficiency, the amount of slow-wave sleep, REM sleep, as well as REM sleep latency, all reduce with aging (Ohayon et al., 2004). Sleep plays an important role in learning, because in sleep new memories are consolidated to long-term memory (Klinzing et al., 2019). During sleep, a person also simulates events that improve learning and memory.

Several studies have indicated that disturbances in both sleep quality and duration may impact cognition in older adults (Yaffe et al., 2014) (Wardle-Pinkston et al., 2019). Most of these studies are cross-sectional and variable-oriented and have shown mixed results. For example, in a study among US participants aged 65–80 years, self-reported sleep quality (based on the Pittsburgh Sleep Quality Index) and several objective measurements of cognition were applied, and it was reported that poorer sleep quality was associated with poorer cognition (Nebes et al., 2009). In another study on US adults aged 70 years and over, with self-reported sleep quality (based on the Medical Outcomes Study Sleep scale) and objectively measured cognition (based on the Blessed Information-Memory-Concentration test), found that poor sleep quality was associated with cognition problems (Zimmerman et al., 2012). A cross-sectional cohort study on older (aged 70 years and over) US community-dwelling men similarly showed associations between self-reported (based on the Pittsburgh Sleep Quality Index and Epworth Sleepiness Scale), objectively measured (measured by wrist actigraphy), sleep disturbances and objectively measured cognitive decline (based on the Trail Making Test B and Modified Mini-Mental State tests) (Blackwell et al., 2014). However, other studies have not found any associations between self-reported insomnia and objective cognitive measurements (Merlino et al., 2010; Saint Martin et al., 2012). In an Italian interview study on 65+-year-old participants, subjective sleep disturbances as well as self-reported daytime sleepiness and cognition (based on the Mini Mental State Examination and Global Deterioration Scale) were measured, and no association between insomnia and cognition decline or dementia was found, but excessive daytime sleepiness was related to dementia (Merlino et al., 2010). In a French study on 65+ year olds, self-reported sleep quality (based on the Pittsburgh Sleep Quality Index) was not associated either with subjective or objective measurements of cognitive function (Saint Martin et al., 2012).

There are only a few prospective studies investigating the associations between insomnia and cognition (Cricco et al., 2001; Foley et al., 2001; Sha et al., 2019; Suh et al., 2018; Virta et al., 2013). In a 3-year follow-up among 65+ year olds, an association between insomnia (based on self-rated trouble falling asleep or waking up) and cognitive decline (based on the Short Portable Mental Status Questionnaire) was found in men but not in women (Cricco et al., 2001). In another 3-year follow-up study (Japanese-American men aged 71 to 93 years), there was no association between insomnia (based on a self-rated questionnaire regarding trouble falling asleep, early morning awakening and daytime sleepiness) and cognitive decline (based on the Cognitive Abilities Screening Instrument), but there was an association between daytime sleepiness and cognitive decline (Foley et al., 2001). In a longer cohort follow-up study (the Finnish Twin Cohort, aged 65 years or over), both long and short sleep and poor sleep quality (based on a self-rated duration and quality questionnaire) were found to be associated with cognitive decline (based on a telephone interview) (Virta et al., 2013). Previous studies have also investigated connections between sleep duration and cognition (Devore et al., 2014; Zitser et al., 2020). A study on 70+-year-old nurses found an association between both short and long sleep and poorer cognition (Devore et al., 2014). The Whitehall study on British civil servants did not find any association (cognitive estimation was based on white-matter volume in magnetic resonance imaging and questionnaire tests) (Zitser et al., 2020). Instead, the recent Whitehall study II on 25-year follow-up found an association between short sleep duration in midlife and an increased risk of dementia in later life (Sabia et al., 2021). However, to our best knowledge, there are no studies that have longitudinally investigated the associations between insomnia symptom trajectories and cognitive function in a time frame from working age to retirement and older age.

The objective of our study is to investigate how insomnia symptoms may affect cognitive function (memory, learning, and concentration) in the time frame from working age to retirement. We additionally distinguish between disability retirement and statutory retirement. We use a person-oriented approach to identify sleep trajectories that can lead to cognitive problems in these groups.

## Methods

### Data

Our data consist of prospective survey data from the Helsinki Health Study (Lahelma et al., 2013). The baseline survey questionnaire was sent in 2000 to 2002 to 40- to 60-year-old employees (in sectors of general local administration, health and social care, education and culture, public transport, and technical services) of the City of Helsinki, the capital of Finland (*N* = 8960, response rate 67%). Follow-up surveys were collected in 2007 (response rate 83%), 2012 (response rate 79%), and 2017 (response rate 82%). The present study focuses on insomnia symptoms and their association with cognitive function of retired older adults. In 2017, 55% of respondents were on statutory or disability retirement and almost all of those (99%) had provided information of their cognitive function (i.e., memory, concentration, and learning ability). Insomnia symptoms were included from all phases if available. Our final sample included those who met these criteria and whose retirement age was known. The final analytic sample consisted of 3748 retired persons aged 55–77 years in 2017 (80% women). We excluded participants who were still working (*n* = 2681) and those who had retired or exited employment for reasons other than disability or statutory retirement (*n* = 403).

### Measurement

#### Sleeping Habits

##### Sleeping Habits Were Self-Assessed at Each Phase.

Insomnia symptoms were measured with four self-rated questions according to the Jenkins Sleep Questionnaire (JSQ) (Jenkins et al., 1988; Lallukka et al., 2011). It was asked, how often in the past month (four weeks) did you: “Have trouble falling asleep?,” “Wake up several times per night?,” “Have trouble staying asleep (including waking far too early)?,” “Wake up after your usual amount of sleep feeling tired and worn out?.” A time-dependent score was created for each phase. Scores ranged from 0 to 5 (with corresponding responses from “not at all,” “1–3 days,” “4–7 days,” “8–14 days,” “15–21 days,” “22–28 days”) were summed up to a total score ranging from 0 to 20. This score was used to estimate sleep trajectories. Cronbach’s alpha was 0.84; it indicated good internal consistency and that it was acceptable to construct a reliable total score.

Sleep duration was measured by asking: how many hours do you sleep on average per day in whole hours during weekdays: “5 hours or less,” “6 hours,” “7 hours,” “8 hours,” “9 hours,” and “10 hours or more.” The answers were categorized into three groups (“short <6 hours,” “mid-range 6–8 hours,” “long >8 hours”) with the assumption that mid-range is the normal sleep duration and the others indicate problems (Kronholm et al., 2009; Lallukka et al., 2018).

#### Cognitive Function

Three aspects of cognitive function—memory, concentration, and learning—were assessed using the following three items: “How well my memory works,” “How well embracing and learning new things goes for me,” “Normally I can concentrate on something.” Responses were given on a five-point scale (“very poorly,” “poorly,” “satisfactorily,” “well,” “very well”). In our sample, the numbers of observations in the highest and lowest category were small. Therefore, these groups were categorized as good (includes “well” and “very well”), mild (includes “satisfactorily”) and poor (includes “very poorly” and “poorly”). This instrument is included in TOIMIA-database which is maintained by the Finnish Institute for Health and Welfare (THL) (The Finnish Institute for Health and Welfare, (THL) ).

#### Covariates

##### Covariates Were Measured at the Last Phase, in 2017.

Of sociodemographic factors, we included age (as a continuous variable), retirement age (as a continuous variable), retirement classification (statutory retirement, disability retirement), gender (male, female), education (basic, secondary, higher), and occupational class. Occupational class was obtained from the employer’s personnel register for those with consent to link survey with register data and completed from the self-reported occupation titles for the rest. Categories were “managers and professionals” [e.g., doctors, teachers], “semi-professionals” [e.g., nurses, technicians], “routine non-manual workers” [e.g., care workers], and “manual workers” [e.g., cleaners, transport workers].

Of health-related behaviors, we included smoking status (no, yes), binge drinking status (no, yes = males: drinking over six units of alcohol at least once a week, females: at least once a month), body mass index (BMI; recommended healthy weight, overweight >25), physical activity scored as metabolic equivalent rate MET (physical inactive MET <14, physical active MET ≥ 14) (Ainsworth et al., 1993; VanItallie, 1996).

Of health factors, we included current pain status (no, yes). The following physician-diagnosed conditions were included: cardiovascular diseases (hypertension, high cholesterol, diabetes), pulmonary diseases (COPD, asthma), sleep apnea, and psychiatric diseases (depression, anxiety disorder, other mental disorder).

### Statistical Analyses

We first computed descriptive cross tabulations to investigate the bivariate relationships of retirement classification, gender, outcomes, and covariates. χ2 tests were performed. We did not have sufficient statistical power for gender-stratified analyses; the rest of the analysis was therefore conducted using pooled data. For outcomes and diseases, missing data were analyzed as its own category. For covariates, missing data were rare so that the missing category was merged with other groups following the best practice of the field (in education to basic, in occupational class to manual workers, in marital status to other, in smoking to no, in binge drinking to no, in BMI to normal weight, in MET to physically active). Overall, we did not identify in our descriptive tables any bias in missing data regarding the different groups.

The trajectories of sleep problems were explored using latent class linear mixed models with R package LCMM (Proust-Lima et al., 2017). The method was applied to find latent groups from the participants who have similar insomnia symptoms trajectories over the study period. We used the total score of insomnia symptoms and the sleep duration classification as an outcome of the function over the time periods. The time axis was modified to present the time difference between the age at the particular phase and the actual retirement age of the participant. Retirement age was defined as the zero-time point. This also provides us information on how sleep curves behave before and after retirement (i.e., in neighborhood of the retirement). The data were tested and fitted as two to four latent classes and first-, second-, and third-degree polynomials (online Supplementary Figures S1 and S2). Time was applied as a random effect. We chose the best model according to the Bayesian information criteria (BIC), Akaike Information Criteria (AIC), average posterior probability of assignment criteria (APPA), odds of correct classification criteria (OCC), the size of the classes, and interpretation of results according to our best understanding of the phenomenon (online Supplementary Tables S1 and S2). Based on the highest probability of these latent classes, the participants were assigned into a trajectory class. The model indicates good fit, high average group membership probability, and we can conclude that trajectory analysis assigned distinct groups very well.

We cross-tabulated these latent groups with cognitive function and examined those associations with χ2. We then used multinomial logistic regression models with these trajectory groups to describe effects of outcomes and covariates. We tested five different models: model 1 adjusted for age and gender, model 2 adjusted for other socio-demographic covariates, model 3 adjusted for health-related behavior, model 4 adjusted for health factors, and model 5 was the full model with all covariates. The results are presented as odds ratios (OR) and their 95% confidence intervals (CI).

All analyses were conducted using the R program, version 3.6.3 (R Core Team ).

## Results

Background characteristics of the study participants are shown in Table 1. In our sample, we categorized participants into two groups: statutory retirement (*N* = 3295, mean retirement age 63.0) and disability retirement (*N* = 453, mean retirement age 57.7). For statutory retirees, 2.6% experienced their memory as poor, 6.0% their learning as poor, and 2.6% their concentration as poor. For those who had retired due to disability reasons, the corresponding proportions were 6.2%, 11.9%, and 8.8%, respectively. Men reported more cognitive problems than women in both retirement groups. Low occupational class, poor health-related behaviors, and poor health were strongly associated with disability retirement. Pain problems and psychiatric diseases were much more common in those who had retired for disability reasons than in those who had retired at the statutory retirement age (pain problems 64.6% vs 36.4%, psychiatric diseases 41.5% vs 9.1%, respectively).

**Table 1.** Background characteristics of the study population.

	Retirement classification			Statutory retirement			Disability retirement		
	Statutory	Disability	P-value	Male	Female	P-value	Male	Female	P-value
	N = 3295 (col%)	N = 453 (col%)		N = 696 (col%)	N = 2599 (col%)		N = 61 (col%)	N = 392 (col%)	
**Outcomes - Cognitive function**									
Memory									
Good	2170 (65.9)	236 (52.1)	<.001	402 (57.8)	1768 (68.0)	<.001	29 (47.5)	207 (52.8)	.756
Mild	1025 (31.1)	183 (40.4)		266 (38.2)	759 (29.2)		27 (44.3)	156 (39.8)	
Poor	87 (2.6)	28 (6.2)		24 (3.4)	63 (2.4)		4 (6.6)	24 (6.1)	
No answer	13 (0.4)	6 (1.3)		4 (0.6)	9 (0.3)		1 (1.6)	5 (1.3)	
Learning				266 (38.2)	759 (29.2)				
Good	1539 (46.7)	170 (37.5)	<.001	289 (41.5)	1250 (48.1)	.00304	22 (36.1)	148 (37.8)	.932
Mild	1527 (46.3)	223 (49.2)		349 (50.1)	1178 (45.3)		29 (47.5)	194 (49.5)	
Poor	198 (6.0)	54 (11.9)		53 (7.6)	145 (5.6)		8 (13.1)	46 (11.7)	
No answer	31 (0.9)	6 (1.3)		5 (0.7)	26 (1.0)		2 (3.3)	4 (1.0)	
Concentration									
Good	2249 (68.3)	242 (53.4)	<.001	451 (64.8)	1798 (69.2)	.0744	25 (41.0)	217 (55.4)	.146
Mild	929 (28.2)	167 (36.9)		220 (31.6)	709 (27.3)		25 (41.0)	142 (36.2)	
Poor	85 (2.6)	40 (8.8)		18 (2.6)	67 (2.6)		8 (13.1)	32 (8.2)	
No answer	32 (1.0)	4 (0.9)		7 (1.0)	25 (1.0)		3 (4.9)	1 (0.3)	
**Sociodemographic - covariates**									
Gender									
Male	696 (21.1)	61 (13.5)	<.001	696 (100)	0 (0)	<.001	61 (100)	0 (0)	<.001
Female	2599 (78.9)	392 (86.5)		0 (0)	2599 (100)		0 (0)	392 (100)	
Retirement age (SD)	63.0 (2.83)	57.7 (4.60)	<.001	63.0 (2.97)	63.1 (2.79)	<.001	58.0 (4.17)	57.6 (4.66)	<.001
Age at final follow-up (SD)	70.0 (3.95)	64.2 (5.43)	<.001	70.3 (4.13)	69.9 (3.90)	<.001	65.9 (6.20)	63.9 (5.26)	<.001
Retirement period years (SD)	6.96 (4.91)	6.53 (5.21)	<.001	7.29 (4.87)	6.87 (4.91)	<.001	7.87 (5.46)	6.32 (5.14)	<.001
Education									
Basic	1457 (44.2)	226 (49.9)	<.001	280 (40.2)	1177 (45.3)	<.001	33 (54.1)	193 (49.2)	.654
Secondary	853 (25.9)	165 (36.4)		149 (21.4)	704 (27.1)		19 (31.1)	146 (37.2)	
Higher	985 (29.9)	62 (13.7)		267 (38.4)	718 (27.6)		9 (14.8)	53 (13.5)	
Occupational class									
Managers and professionals	1192 (36.2)	74 (16.3)	<.001	356 (51.1)	836 (32.2)	<.001	12 (19.7)	62 (15.8)	<.001
Semi-professionals	522 (15.8)	92 (20.3)		129 (18.5)	393 (15.1)		14 (23.0)	78 (19.9)	
Routine non-manual workers	1105 (33.5)	189 (41.7)		43 (6.2)	1062 (40.9)		10 (16.4)	179 (45.7)	
Manual workers	476 (14.4)	98 (21.6)		168 (24.1)	308 (11.9)		25 (41.0)	73 (18.6)	
Marital status									
Other	1258 (38.2)	191 (42.2)	.114	149 (21.4)	1109 (42.7)	<.001	22 (36.1)	169 (43.1)	.37
Co-habiting/married	2037 (61.8)	262 (57.8)		547 (78.6)	1490 (57.3)		39 (63.9)	223 (56.9)	
**Health-related behaviors - covariates**									
Smoking									
Yes	294 (8.9)	78 (17.2)	<.001	72 (10.3)	222 (8.5)	.159	15 (24.6)	63 (16.1)	.145
No	3001 (91.1)	375 (82.8)		624 (89.7)	2377 (91.5)		46 (75.4)	329 (83.9)	
Binge drinking									
No	2971 (90.2)	401 (88.5)	.313	585 (84.1)	2386 (91.8)	<.001	52 (85.2)	349 (89.0)	.518
Yes	324 (9.8)	52 (11.5)		111 (15.9)	213 (8.2)		9 (14.8)	43 (11.0)	
Body mass index									
Recommended healthy weight	1392 (42.2)	127 (28.0)	<.001	273 (39.2)	1119 (43.1)	.0761	13 (21.3)	114 (29.1)	.27
Overweight (BMI >=25)	1903 (57.8)	326 (72.0)		423 (60.8)	1480 (56.9)		48 (78.7)	278 (70.9)	
Metabolic Equivalent									
Physically inactive (MET<14)	751 (22.8)	174 (38.4)	<.001	180 (25.9)	571 (22.0)	.0338	29 (47.5)	145 (37.0)	.151
Physically active (MET>=14)	2544 (77.2)	279 (61.6)		516 (74.1)	2028 (78.0)		32 (52.5)	247 (63.0)	
**Health factors - covariates**									
Pain									
No	1922 (58.3)	136 (30.0)	<.001	447 (64.2)	1475 (56.8)	<.001	20 (32.8)	116 (29.6)	.211
Yes	1199 (36.4)	292 (64.5)		209 (30.0)	990 (38.1)		35 (57.4)	257 (65.6)	
No answer	174 (5.3)	25 (5.5)		40 (5.7)	134 (5.2)		6 (9.8)	19 (4.8)	
Disease - cardiovascular									
Yes	2054 (62.3)	295 (65.1)	.0578	453 (65.1)	1601 (61.6)	.0779	43 (70.5)	252 (64.3)	.416
No	800 (24.3)	88 (19.4)		167 (24.0)	633 (24.4)		12 (19.7)	76 (19.4)	
No answer	441 (13.4)	70 (15.5)		76 (10.9)	365 (14.0)		6 (9.8)	64 (16.3)	
Disease - pulmonary									
Yes	403 (12.2)	84 (18.5)	<.001	78 (11.2)	325 (12.5)	.00377	10 (16.4)	74 (18.9)	.876
No	1705 (51.7)	215 (47.5)		399 (57.3)	1306 (50.3)		29 (47.5)	186 (47.4)	
No answer	1187 (36.0)	154 (34.0)		219 (31.5)	968 (37.2)		22 (36.1)	132 (33.7)	
Disease - psychiatric									
Yes	301 (9.1)	188 (41.5)	<.001	40 (5.7)	261 (10.0)	<.001	22 (36.1)	166 (42.3)	.0644
No	1784 (54.1)	150 (33.1)		420 (60.3)	1364 (52.5)		28 (45.9)	122 (31.1)	
No answer	1210 (36.7)	115 (25.4)		236 (33.9)	974 (37.5)		11 (18.0)	104 (26.5)	
Disease - sleep apnea									
Yes	153 (4.6)	54 (11.9)	<.001	57 (8.2)	96 (3.7)	<.001	14 (23.0)	40 (10.2)	.0166
No	1826 (55.4)	233 (51.4)		401 (57.6)	1425 (54.8)		28 (45.9)	205 (52.3)	
No answer	1316 (39.9)	166 (36.6)		238 (34.2)	1078 (41.5)		19 (31.1)	147 (37.5)	

SD standard deviation.

P-value from the chi-square test.

MET metabolic equivalent.

BMI body mass index.

**Figure 1.**
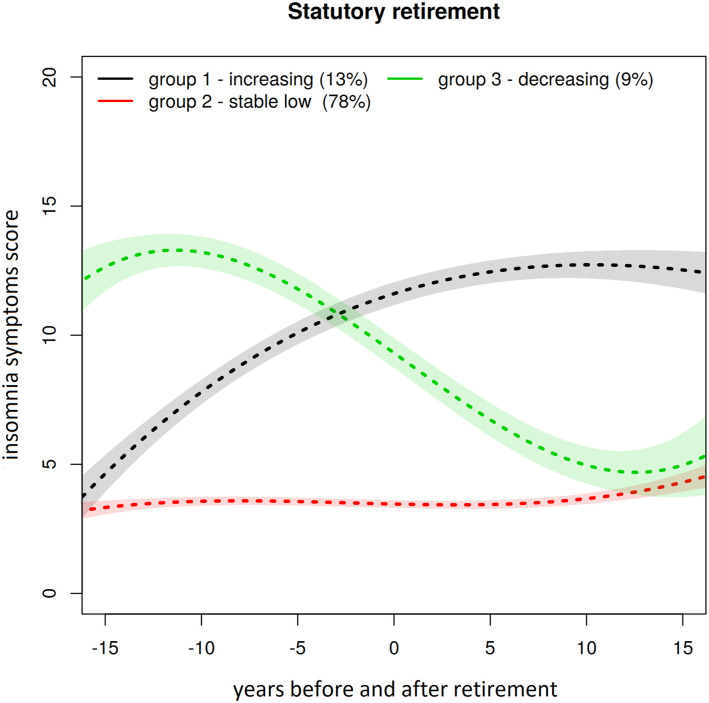
Estimated insomnia symptoms trajectories, confidence intervals, and group sizes in statutory retirement (0 = retirement year).

**Figure 2.**
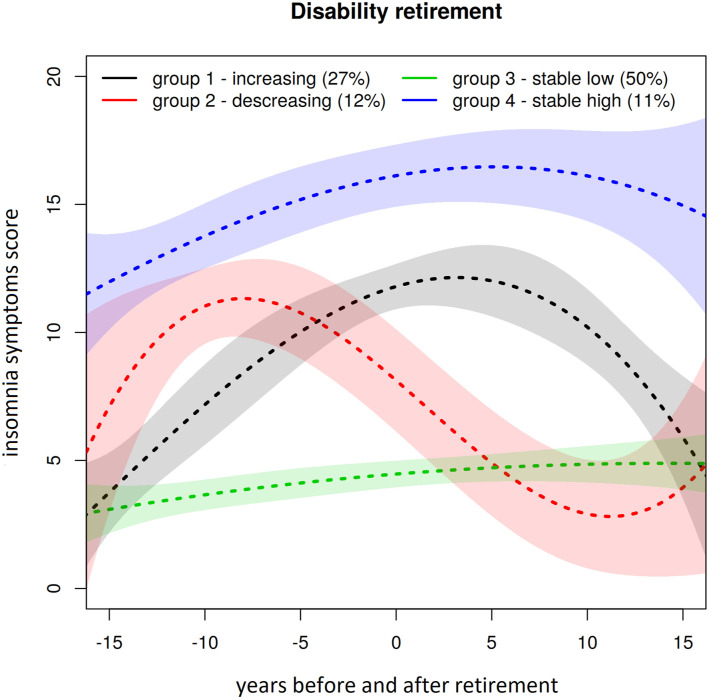
Estimated insomnia symptoms trajectories, confidence intervals, and group sizes in disability retirement (0 = retirement year).

We identified three latent group trajectories of insomnia symptoms among statutory retirees over the follow-up period (Figure 1). The groups were stable low (78%), decreasing (9%), and increasing (13%). Among those who had retired for disability reasons, we identified four latent group trajectories (Figure 2): stable low (50%), decreasing (12%), increasing (27%), and stable high (11%). We also tested sleep duration trajectories, but our sample sizes were too small to distinguish clear latent groups. Table 2 shows the associations between the latent groups and cognitive function. Statutory retirees in the stable low trajectory group experienced their memory (1%), learning (4%), concentration (1%) as being poor less often than those who were on decreasing or increasing trajectories (5%/10%/5% and 9%/14%/9%, respectively). Among those on disability retirement, participants in the stable low trajectory group experienced their memory (4%), learning (8%), concentration (5%) as being poor less often than those who were on decreasing, increasing or stable high trajectories (2%/13%/4%, 8%/16%/16% and 16%/22%/14%, respectively).

**Table 2.** Sleep trajectories and cognition by retirement categories.

Statutory retirement	Cognitive function - Memory				Cognitive function - Learning				Cognitive function - Concentration			
	Good	Mild	Poor	P-value	Good	Mild	Poor	P-value	Good	Mild	Poor	P-value
	(N = 2170)	(N = 1025)	(N = 87)		(N = 1539)	(N = 1527)	(N = 198)		(N = 2249)	(N = 929)	(N = 85)	
Sleep trajectory												
group 1 - increasing	48%	44%	9%	<.001	29%	56%	14%	<.001	52%	39%	9%	<.001
group 2 - stable low	70%	29%	1%		51%	44%	4%		72%	26%	1%	
group 3 - decreasing	60%	35%	5%		36%	52%	10%		59%	35%	5%	

Disability retirement	Cognitive function - Memory				Cognitive function - Learning				Cognitive function - Concentration			
	Good	Mild	Poor	P-value	Good	Mild	Poor	P-value	Good	Mild	Poor	P-value
	(N = 236)	(N = 183)	(N = 28)		(N = 170)	(N = 223)	(N = 54)		(N = 242)	(N = 167)	(N = 40)	
Sleep trajectory												
group 1 - increasing	43%	49%	8%	.00359	25%	60%	16%	.00114	50%	34%	16%	.00205
group 2 - decreasing	53%	45%	2%		38%	51%	13%		57%	42%	4%	
group 3 - stable low	59%	37%	4%		45%	47%	8%		59%	36%	5%	
group 4 - stable high	52%	32%	16%		42%	36%	22%		38%	48%	14%	

In Table 3, odds ratios of the five different models are shown to compare associations between latent trajectory groups and cognitive function. In age- and gender-adjusted models among statutory retirees, when comparing the increasing insomnia symptoms trajectory to the reference stable-low group, the ORs were: poor memory OR = 10.3 (95% CI 6.3–16.9) and mild memory OR = 2.4 (95% CI 1.9–3.0). Respectively, poor learning OR = 6.4 (95% CI 4.4–9.3) and mild learning OR = 2.3 (95% CI 1.8–2.9). Respectively, poor concentration OR=9.5 (95% CI 5.8–15.5) and mild concentration OR = 2.2 (95% CI 1.7–2.7). When the same comparison was made between the trajectory group of decreasing insomnia symptoms and the reference group, the ORs were: poor memory OR = 5.4 (95% CI 2.9–10.2), mild memory OR = 1.6 (1.2–2.1), poor learning OR = 4.4 (2.8–7.0), mild learning OR = 1.9 (1.4–2.5), poor concentration OR=5.0 (2.6–9.6), and mild concentration OR = 1.8 (1.4–2.3). In the fully adjusted model, ORs were roughly one-fifth smaller and this is explained mostly by health factors. Sociodemographic and health-related behaviors had only a slight effect on the associations. Among those on disability retirement, we could not estimate ORs due to small numbers.

**Table 3.** Multinomial logistic regression models by retirement categories to compare associations between latent insomnia symptoms trajectory groups and cognitive function.

	Cognitive function - Memory		Cognitive function - Learning		Cognitive function - Concentration	
	Mild	Poor	Mild	Poor	Mild	Poor
Statutory retirement	OR (95% CI)	OR (95% CI)	OR (95% CI)	OR (95% CI)	OR (95% CI)	OR (95% CI)
Model 1 - age and gender adjusted						
group 1 - increasing	2.4 (1.9, 3.0)	10.3 (6.3, 16.9)	2.3 (1.8, 2.9)	6.4 (4.4, 9.3)	2.2 (1.7, 2.7)	9.5 (5.8, 15.5)
group 3 - decreasing	1.6 (1.2, 2.1)	5.4 (2.9, 10.2)	1.9 (1.4, 2.5)	4.4 (2.8, 7.0)	1.8 (1.4, 2.3)	5.0 (2.6, 9.6)
Model 2 - sociodemographic covariates adjusted						
group 1 - increasing	2.3 (1.8, 2.8)	9.4 (5.8, 15.3)	2.3 (1.8, 2.9)	5.9 (4.1, 8.6)	2.2 (1.7, 2.7)	9.3 (5.7, 15.2)
group 3 - decreasing	1.4 (1.1, 1.8)	4.5 (2.4, 8.5)	1.7 (1.3, 2.2)	3.6 (2.3, 5.6)	1.6 (1.2, 2.1)	4.5 (2.4, 8.6)
Model 3 - health-related behaviors covariates adjusted						
group 1 - increasing	2.2 (1.8, 2.7)	8.9 (5.5, 14.6)	2.2 (1.7, 2.7)	5.7 (3.9, 8.2)	2.0 (1.6, 2.5)	8.9 (5.4, 14.5)
group 3 - decreasing	1.4 (1.1, 1.8)	4.5 (2.4, 8.3)	1.7 (1.3, 2.2)	3.5 (2.2, 5.5)	1.6 (1.2, 2.1)	4.5 (2.3, 8.5)
Model 4 - health factors covariates adjusted						
group 1 - increasing	1.9 (1.5, 2.4)	7.7 (4.6, 12.8)	2.0 (1.6, 2.6)	4.7 (3.2, 6.9)	1.9 (1.5, 2.3)	6.7 (4.0, 11.2)
group 3 - decreasing	1.2 (0.9, 1.6)	3.4 (1.8, 6.5)	1.5 (1.2, 2.0)	2.8 (1.8, 4.5)	1.4 (1.1, 1.8)	3.0 (1.6, 5.9)
Model 5 - fully adjusted						
group 1 - increasing	2.1 (1.6, 2.6)	8.6 (5.1, 14.6)	2.2 (1.7, 2.8)	5.3 (3.6, 8.0)	2.0 (1.6, 2.5)	7.2 (4.3, 12.3)
group 3 - decreasing	1.4 (1.0, 1.8)	4.0 (2.1, 7.8)	1.7 (1.3, 2.2)	3.6 (2.2, 5.8)	1.5 (1.2, 2.0)	3.5 (1.8, 6.9)
Disability retirement	OR (95% CI)	OR (95% CI)	OR (95% CI)	OR (95% CI)	OR (95% CI)	OR (95% CI)
Model 1 - age and gender adjusted						
group 1 - increasing	1.8 (1.1, 2.9)	2.7 (1.0, 7.1)	2.4 (1.4, 3.9)	3.9 (1.8, 8.4)	1.2 (0.7, 1.9)	3.9 (1.7, 8.7)
group 2 - decreasing	1.3 (0.7, 2.4)	0.5 (0.1, 4.1)	1.3 (0.7, 2.5)	2.1 (0.8, 5.7)	1.2 (0.6, 2.2)	0.7 (0.1, 3.2)
group 4 - stable high	1.0 (0.5, 1.9)	4.3 (1.5, 12.2)	0.9 (0.4, 1.7)	3.3 (1.3, 8.2)	2.1 (1.1, 4.1)	3.8 (1.3, 11.2)
Model 2 - sociodemographic covariates adjusted						
group 1 - increasing	1.8 (1.1, 2.8)	2.9 (1.1, 7.7)	2.3 (1.4, 3.8)	3.7 (1.7, 8.1)	1.2 (0.7, 1.9)	3.8 (1.7, 8.6)
group 2 - decreasing	1.3 (0.7, 2.4)	0.5 (0.1, 4.2)	1.3 (0.7, 2.5)	2.0 (0.7, 5.5)	1.2 (0.6, 2.2)	0.8 (0.2, 3.7)
group 4 - stable high	0.9 (0.5, 1.9)	4.5 (1.6, 12.8)	0.8 (0.4, 1.6)	3.0 (1.2, 7.3)	2.1 (1.1, 4.2)	4.3 (1.5, 12.7)
Model 3 - health-related behaviors covariates adjusted						
group 1 - increasing	1.8 (1.1, 2.8)	2.8 (1.1, 7.3)	2.3 (1.4, 3.8)	3.6 (1.7, 7.9)	1.2 (0.7, 1.9)	3.8 (1.7, 8.5)
group 2 - decreasing	1.3 (0.7, 2.4)	0.5 (0.1, 4.3)	1.3 (0.7, 2.5)	2.0 (0.7, 5.5)	1.2 (0.7, 2.3)	0.8 (0.2, 3.7)
group 4 - stable high	0.9 (0.5, 1.9)	4.4 (1.6, 12.7)	0.8 (0.4, 1.6)	3.0 (1.2, 7.2)	2.2 (1.1, 4.3)	4.3 (1.5, 12.6)
Model 4 - health factors covariates adjusted						
group 1 - increasing	1.5 (0.9, 2.5)	1.9 (0.7, 5.3)	2.2 (1.3, 3.7)	3.0 (1.3, 6.9)	1.1 (0.6, 1.8)	3.5 (1.5, 8.2)
group 2 - decreasing	1.1 (0.6, 2.1)	0.4 (0.0, 3.5)	1.2 (0.6, 2.3)	1.9 (0.7, 5.3)	1.2 (0.6, 2.2)	0.7 (0.1, 3.5)
group 4 - stable high	0.7 (0.3, 1.4)	2.3 (0.7, 7.2)	0.7 (0.3, 1.4)	1.9 (0.7, 5.0)	1.8 (0.9, 3.6)	3.1 (1.0, 9.9)
Model 5 - fully adjusted						
group 1 - increasing	1.5 (0.9, 2.6)	1.8 (0.6, 5.7)	2.2 (1.3, 3.9)	3.0 (1.3, 7.1)	1.1 (0.6, 1.8)	3.4 (1.4, 8.5)
group 2 - decreasing	1.2 (0.6, 2.3)	0.4 (0.0, 3.4)	1.3 (0.6, 2.6)	2.0 (0.7, 5.8)	1.0 (0.5, 2.0)	0.6 (0.1, 3.2)
group 4 - stable high	0.7 (0.3, 1.5)	2.4 (0.7, 8.4)	0.7 (0.3, 1.5)	2.1 (0.7, 5.9)	1.7 (0.8, 3.7)	2.9 (0.9, 9.9)

SD standard deviation.

OR, odds ratio; CI, confidence interval. Model 2 - sociodemographic covariates adjusted: age, gender, retirement age, education, occupation class, marital status.

Model 3 - health-related behaviors covariates adjusted: smoking, binge drinking, body mass index, metabolic equivalent. Model 4 - health factors covariates adjusted: pain, cardiovascular diseases, pulmonary diseases, psychiatric diseases, sleep apnea.Model 5 - fully adjusted: all covariates from models 2-4.

## Discussion

### Main Findings

The purpose of this study was to identify trajectories of insomnia symptoms among aging employees, and investigate the associations of these trajectories with memory, concentration, and learning ability after retirement across a 15- to 17-year follow-up.

We found three latent group trajectories of insomnia symptoms (stable low, decreasing and increasing) among statutory retirees. Among disability retirees, we identified four latent group trajectories (stable low, decreasing, increasing, and stable high). Findings indicated that belonging to the trajectory of severe insomnia symptoms was associated with worse cognitive function among statutory retirees. About one-fifth of the increased risk of poor cognitive function in trajectories was explained by health factors among statutory retirees. Sociodemographic factors and health-related behaviors had a smaller effect.

### Interpretation

Our study showed associations between self-reported insomnia symptoms and self-reported cognition. In our study, there were three to four trajectory groups according to when insomnia symptoms occur with respect to retirement. Our person-oriented approach characterized these subgroups without a priori assumptions. Most of the previous studies have used variable-based methods in which predefined cut-off points are used and subjective bias is possible (Cricco et al., 2001; Foley et al., 2001; Suh et al., 2018; Virta et al., 2013). The person-oriented approach also better observes the development of individuals over time, finds latent classes and focuses on the process itself.

There are many mechanisms that can explain how sleep influences cognitive function, for example, glymphatic metabolite clearance activation at night, frontal and medial temporal lobe restoration regarding slow wave and REM sleep and memory consolidation in slow-wave sleep (Scullin & Gao, 2018). Many studies have used a cross-sectional approach, which does not consider a long-term effect of insomnia problems (Blackwell et al., 2014; Merlino et al., 2010; Nebes et al., 2009; Saint Martin et al., 2012; Zimmerman et al., 2012). These studies can demonstrate that insomnia is associated with cognitive function but our study can estimate the effect of insomnia symptoms before changes in cognitive function occur.

Our results showed that insomnia symptoms already in working age can increase the risk of cognitive decline in retirement age. The analysis showed that increased sleeping complaints were related to more severe problems in subjective cognitive function. In addition, sleeping trajectories suggested that if problems improve over the years, cognitive function was also rated better in retirement age. The group of stable-low sleeping problems had better cognitive function in the future than those who had at least some insomnia symptoms. If the area under the curve in Figure 1 is interpreted, the cumulative effect is slightly higher in the increasing than in the decreasing trajectory. However, odds ratios are double in the increasing group, which implies that aging may contribute more to background mechanisms which lead to cognitive decline, if linearity is assumed.

Those on disability retirement experienced more cognition problems compared to statutory retirees. They also had more insomnia symptoms. The different sleep trajectories of disability retirees did not increase risks at the same level compared to the more problematic sleeping groups in statutory retirees. The risk of having poorer cognitive function was increased as a whole, however, so sleeping habits play a smaller role.

In our results, we showed that health factors partially explained the odds of poorer cognitive function. We adjusted our models for many well-known risk factors of dementia: age, high blood pressure, high cholesterol, obesity, diabetes, depression, low education level, and low level of physical activity; only genetic factors such as ApoE were excluded (Baumgart et al., 2015). After adjustments, there were still significant effects that were explained by insomnia symptoms. We suggest that longstanding insomnia symptoms should also be considered as risk factors of cognitive decline.

In clinical practice, we are interested in whether the decline in subjective cognitive function suggests that there is a latent memory disease such as Alzheimer’s disease, mild cognitive impairment, or vascular dementia, although the scientific evidence is controversial (Iliffe & Pealing, 2010; Jonker et al., 2000; Mitchell, 2008; Snitz et al., 2015). With early detection of insomnia symptoms, it might be possible to prevent cognitive decline and potentially slow the development of memory diseases like Alzheimer’s disease. In sleep, the human body removes tau protein, a microtubule-associated protein, which forms neuro-fibrilla lesions in, for example, Alzheimer’s disease (Benedict et al., 2020; Lucey et al., 2019). As a consequence, sleep problems may increase this protein and can raise the risk of dementia. Sleep-quality problems are also associated with an accumulation of β-amyloid and this may increase the risk of dementia (Brown et al., 2016; Spira et al., 2013).

### Clinical Implications

Further studies need to be conducted to estimate how the treatment of insomnia might prevent cognitive decline. There are many ways to improve sleep, both non-pharmacological and pharmacological. Sleep hygiene consists of sleep-related behaviors, environmental conditions, and other factors that are important to maintain good sleep (Stepanski & Wyatt, 2003). In order to improve sleep, these factors need to be considered. Aerobic physical activity combined with sleep hygiene is also important to improve sleep quality and duration (Reid et al., 2010). In addition, alcohol use affects sleep (Roehrs & Roth, 2001). Small doses of alcohol might help sleep, but excessive alcohol use in particular can interfere with sleep. Cigarette smoking can cause sleep disturbances, and smokers are more likely to have insomnia symptoms (Jaehne et al., 2012; Wetter & Young, 1994). There are also findings suggesting that cognitive psychotherapy (Harvey et al., 2005) and cognitive training (Haimov & Shatil, 2013) may help people suffering from insomnia.

If non-pharmacological techniques fail to work, there might be a need for sleep medications. Sedative hypnotics (for example, benzodiazepines) are widely used for insomnia, but they have many adverse effects such as risk of falls or cognitive decline, especially in older adults (Haimov & Shatil, 2013). It is recommended that benzodiazepines should only be used in the short-term because they cause addiction and tolerance. Melatonin (Ferracioli-Oda et al., 2018) or some antidepressants (Wilson, & Argyropoulos, 2005) would be better for improving sleep quality.

### Strengths and Limitations

There are several strengths in this study. First, we had rich comprehensive longitudinal data of participants over a 15–17-year time period containing four phases. The number of participants was high and the response rates were excellent. Second, the response rates to the cognitive function questions were high. Third, repeatedly collected data on insomnia symptoms were identically measured across four phases, which allowed us to examine the developmental patterns of sleep trajectories and find latent classes. Fourth, our data covered a large number of background variables and almost all well-known risk factors of dementia were included.

Nevertheless, there are also limitations. First, questions of cognitive function were only included in the last phase. Therefore, we cannot follow up the development of memory, learning, and concentration at different time points. However, poor cognitive function is assumed to be relatively rare during earlier phases, when participants were employed. Second, in our data there are no objective memory scores available, for example the Mini-Mental State Examination (MMSE) (Folstein et al., 1975), Montreal Cognitive Assessment (MOCA) (Nasreddine et al., 2005) or Consortium to Establish a Registry for Alzheimer’s Disease (CERAD) (Morris et al., 1989). However, it should be considered that subjective cognitive function estimations can be different from objective measurements. Patients with dementia may suffer anosognosia, therefore cognitive problems may be underreported (Wilson et al., 2016). Third, there are some limitations in the Jenkins sleeping score. Insomnia symptoms were asked only from the last four weeks. However, a three-month period would have been better synchronized with the definition of insomnia. Daytime sleepiness symptoms are also excluded. Fourth, the age of the participants was quite low regarding the development of memory diseases, thus further research could aim to examine how cognitive function develops as the participants age and what the contribution of the course of sleep is to future development of memory diseases. As our cohort ages, we can obtain ICD-10 codes of diagnosed memory diseases from the national health registry. When the sample ages, for example, in 10 years, it is more likely that some of the participants have developed a memory disease. Finally, the cohort is limited to employees of the City of Helsinki at baseline. We thus cannot generalize our results to the entire aging population.

## Conclusions

The present study found three trajectory groups according to when sleeping problems occur regarding statutory retirement: decreasing (insomnia symptoms before retirement age), increasing (insomnia symptoms after retirement), and stable low (good sleep). Among disability retirees, we found one additional group: stable high (insomnia symptoms remain stable regardless of retirement). Insomnia symptoms were associated with worse cognitive function. The effect was more severe if insomnia symptoms increased after retirement.

Our person-oriented approach provides a new perspective on how longstanding insomnia symptoms can cumulatively increase the risk of cognitive problems after retirement. In our data, the variable-like approach also shows that associations between sleeping problems and memory, concentration, and learning ability were stronger in participants who had retired due to disability compared to those who retired at the statutory retirement age.

Early detection of insomnia symptoms already in midlife could be a potential intervention point to improve sleep quality and prevent cognitive decline in later life. These actions might save public funds and improve one’s wellbeing, adding quality-of-life years in the context of aging. Intervention studies are needed, however.

## Supplemental Material

sj-xlsx-1-jah-10.1177_08982643221078740 – Supplemental Material for Trajectories of Insomnia Symptoms Among Aging Employees and Their Associations With Memory, Learning Ability, and Concentration After Retirement - A Prospective Cohort Study (2000–2017) Click here for additional data file. Supplemental Material, sj-xlsx-1-jah-10.1177_08982643221078740 for Trajectories of Insomnia Symptoms Among Aging Employees and Their Associations With Memory, Learning Ability, and Concentration After Retirement - A Prospective Cohort Study (2000–2017) by Antti Etholén, Olli Pietiläinen, Anne Kouvonen, Mirja Hänninen, Ossi Rahkonen and Tea Lallukka in Journal of Aging and Health

sj-xlsx-2-jah-10.1177_08982643221078740 – Supplemental Material for Trajectories of Insomnia Symptoms Among Aging Employees and Their Associations With Memory, Learning Ability, and Concentration After Retirement - A Prospective Cohort Study (2000–2017) Click here for additional data file. Supplemental Material, sj-xlsx-2-jah-10.1177_08982643221078740 for Trajectories of Insomnia Symptoms Among Aging Employees and Their Associations With Memory, Learning Ability, and Concentration After Retirement - A Prospective Cohort Study (2000–2017) by Antti Etholén, Olli Pietiläinen, Anne Kouvonen, Mirja Hänninen, Ossi Rahkonen and Tea Lallukka in Journal of Aging and Health
